# Mammographic texture resemblance generalizes as an independent risk factor for breast cancer

**DOI:** 10.1186/bcr3641

**Published:** 2014-04-08

**Authors:** Mads Nielsen, Celine M Vachon, Christopher G Scott, Konstantin Chernoff, Gopal Karemore, Nico Karssemeijer, Martin Lillholm, Morten A Karsdal

**Affiliations:** 1 Department of Computer Science, University of Copenhagen, Universitetsparken 1, DK-2100 Copenhagen, Denmark; 2 Mayo Clinic College of Medicine, Charlton 6-239|, 200 First Street Southwest, Rochester, MN 55905, USA; 3 Biomediq, Fruebjergvej 3, DK-2100 Copenhagen, Denmark; 4 Department of Radiology, Radboud University Medical Centre Nijmegen, Geert Grooteplein 10, 6525 GA Nijmegen, The Netherlands; 5 Nordic Bioscience, Herlev Hovedgade 207, 2700 Herlev, Denmark

## Abstract

**Introduction:**

Breast density has been established as a major risk factor for breast cancer. We have previously demonstrated that mammographic texture resemblance (MTR), recognizing the local texture patterns of the mammogram, is also a risk factor for breast cancer, independent of percent breast density. We examine if these findings generalize to another population.

**Methods:**

Texture patterns were recorded in digitalized pre-diagnosis (3.7 years) film mammograms of a nested case–control study within the Dutch screening program (S1) comprising of 245 breast cancers and 250 matched controls. The patterns were recognized in the same study using cross-validation to form resemblance scores associated with breast cancer. Texture patterns from S1 were examined in an independent nested case–control study within the Mayo Mammography Health Study cohort (S2) of 226 cases and 442 matched controls: mammograms on average 8.5 years prior to diagnosis, risk factor information and percent mammographic density (PD) estimated using Cumulus were available. MTR scores estimated from S1, S2 and S1 + S2 (the latter two as cross-validations) were evaluated in S2. MTR scores were analyzed as both quartiles and continuously for association with breast cancer using odds ratios (OR) and adjusting for known risk factors including age, body mass index (BMI), and hormone usage.

**Results:**

The mean ages of S1 and S2 were 58.0 ± 5.7 years and 55.2 ± 10.5 years, respectively. The MTR scores on S1 showed significant capability to discriminate cancers from controls (area under the operator characteristics curve (AUC) = 0.63 ± 0.02, *P* <0.001), which persisted after adjustment for PD. S2 showed an AUC of 0.63, 0.61, and 0.60 based on PD, MTR scores trained on S2, and MTR scores trained on S1, respectively. When adjusted for PD, MTR scores of S2 trained on S1 showed an association with breast cancer for the highest quartile alone: OR in quartiles of controls as reference; 1.04 (0.59 to 1.81); 0.95 (0.52 to 1.74); 1.84 (1.10 to 3.07) respectively. The combined continuous model with both PD and MTR scores based on S1 had an AUC of 0.66 ± 0.03.

**Conclusions:**

The local texture patterns associated with breast cancer risk in S1 were also an independent risk factor in S2. Additional textures identified in S2 did not significantly improve risk segregation. Hence, the textural patterns that indicated elevated risk persisted under differences in X-ray technology, population demographics, follow-up time and geography.

## Introduction

Mammographic density [1] has been established as a risk factor of breast cancer. In large epidemiological studies, the highest quartile of mammographic density has shown a four- to sixfold increased risk of breast cancer [2,3] and a substantial fraction of breast cancers may be attributed to this risk factor [4]. It has been hypothesized that mammographic density represents the amount or proportion of fibroglandular tissue present in the breast but the underlying mechanisms of the density and breast cancer association are still uncertain [5]. Recent studies also show that density reductions with tamoxifen use are also associated with decreased breast cancer risk [6]. Thus, mammographic density is both an important risk factor and potential surrogate marker for response to therapy.

Recently, qualifying an objective measure of density, based on quantitative calibrated imaging, measuring the volume percentage of fibroglandular tissue in raw digital mammograms has attracted attention [7], and been associated with breast cancer risk factors [8]. However, several independent studies have shown that also structural components of the density distribution relate independently to breast cancer risk [9-13]. Borrowed from trabecular bone analysis, the fractal dimension [14], as well as several other texture measures, has been suggested. However, which visual patterns of dense structures that are most significantly associated with risk remains to be determined. The mammographic texture resemblance (MTR) [10] does not quantify prespecified properties of the density distribution, but used machine-learning to recognize density distribution patterns in mammograms with known outcome, yielding a density-independent increased breast cancer risk of two- to sixfold.

The underlying mechanisms of heterogeneity that relates to risk can be hypothesized to relate to increased turnover and thereby more disorganized growth of fibrous tissue. It is, however, not *a priori* clear how these disorganized structures will manifest in mammograms and how and whether these patterns can be separated from fibroglandular tissue of normal turnover. A very general approach, recording and recognizing structural components from mammograms of subjects with known outcome has shown very promising results [10,11]. The major problem with such approaches is whether patterns associated with risk persist through changes of population selection, imaging protocol, X-ray technology, digitalization, and so on.

We examine whether structural components and textures were associated with breast cancer risk in two independent [10] studies from different clinics, geographical areas, follow-up times, and with somewhat different demographics.

## Methods

### Study population

Two samples were included in the current study: Study 1 (S1) from the national Dutch screening program, and Study 2 (S2) from the Mayo Mammography Health Study cohort, a screening mammography cohort established at the Mayo Clinic [15]. S1 was collected by the Radboud University, Nijmegen. It included mammograms from 125 screen-detected cases, 120 interval-detected cases and 250 matched controls; all from the same screening units within the biannual Dutch screening program. This cohort was originally selected for the purpose of studying the effect of recall rate [16] and subsequently used for studying the potential of MTR as a marker for breast cancer risk [10]. In accordance with the Helsinki Declaration, women participating in this program were asked to give written informed consent for their data to be used for evaluative purposes. Institutional review board approval was not required. Mammograms were ascertained from 1999 to 2001, two screening rounds prior to diagnosis. Only age and mammographic features were available for this study.

The Mayo Mammography Health Study (MMHS) cohort at the Mayo Clinic in Rochester, Minnesota (MN) was established to examine the association of breast density with breast cancer [13,15]. The MMHS was approved by the Mayo Institutional Review Board. From October 2003 to September 2006, all women scheduled for screening mammography at the Mayo Clinic were invited to participate. Eligible women were residents of Minnesota, Iowa or Wisconsin; age 35+; and had no personal history of breast cancer. A risk factor questionnaire, consent form, and permission to link to tumor registries were obtained. For this study, incident breast cancer was identified through 2009 by linkage to the Mayo Clinic and tri-state cancer registries. A case-cohort of all incident breast cancers and 2,300 randomly selected women (the sub-cohort) were used to examine the association of breast density and breast cancer using the earliest available film mammograms [15]. For this analysis, we matched 442 controls from members of the sub-cohort to 226 cases. Controls from the randomly selected sub-cohort were matched two to one to cases on age and time from the earliest available mammogram to study enrollment/diagnosis date.

### Mammographic measures

Both studies used digitized film mammograms. For S1, the right mediolateral view was digitized on a Vidar scanner (Vidar Systems Corporation, Herndon, VA, USA) providing an image resolution of approximately 1,500 × 2,500 pixels on 12-bit grayscale and size 50 × 50 microns. In S2, four-view mammograms were digitized on the Array 2905 laser film digitizer (Array Corporation, Roden, Netherlands) that similarly has 50 micrometer (limiting) pixel spacing with 12-bit grayscale bit depth.

The breast region was manually outlined as a skin-air curve and a line separating breast tissue from the pectoral muscle. The projected area of the breast region was recorded.

In S1, a trained radiologist estimated percent density (PD) on the right mediolateral oblique (MLO) view using a thresholding approach [17] ignoring subsequent cancer laterality. There was no observed association between cancers and radiological readings of these mammograms by 15 screening radiologists certified by the National Expert and Training Centre for Breast Cancer Screening [16].

In S2, PD was scored by a trained reader on the craniocaudal (CC) or top-down views of the contralateral breast to the cancer (and matched side for controls) using a similar approach as S1, Cumulus [18]. The reader was blinded to cancer outcome in both studies. The MLO view of the same breast was used for MTR scoring.

**Figure 1:**
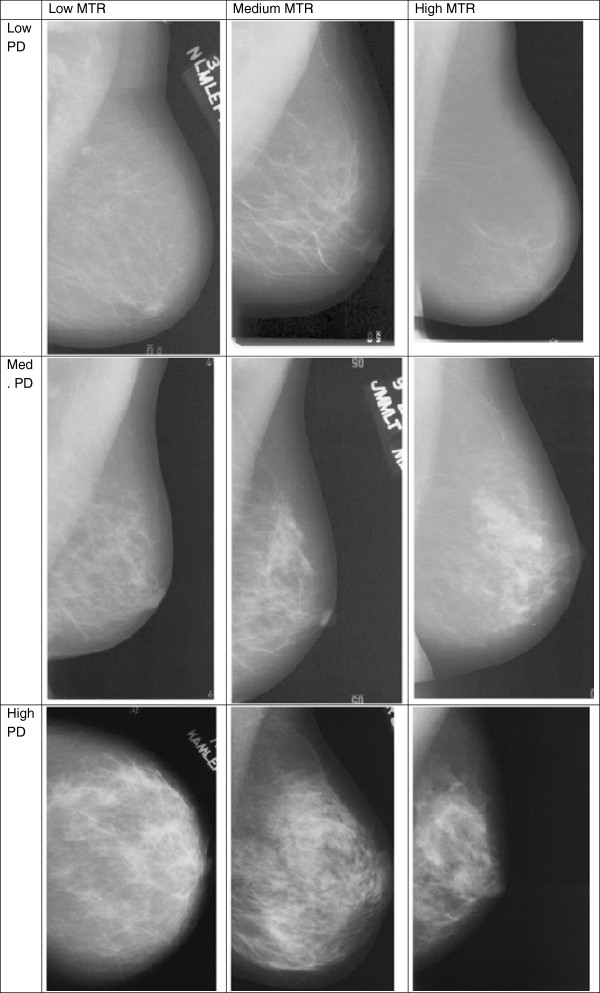
**Mammograms representing each tertile of percent density and mammographic texture resemblance (MTR) scores in S2.** High MTR images seem to have coarser, more large-scale texture.

The MTR scores [10,19] rely on training data where features of the local visual appearance of the mammogram are recorded along with the subject’s case–control status. As in the previous study on S1 [10], each feature vector contained 40 numbers reflecting the attenuation variation in the anterior-posterior direction and orthogonally at length scales of 1 to 8 mm around one single point using the Gaussian scale-space 3-jet [20]. MTR scores are formed by sampling uniformly 20,000 positions and retrieving the training patches of most alike features and cumulating their outcome [19]. Examples of mammograms with increasing MTR score for low-, medium-, and high-density breasts are given in Figure 1, illustrating the higher large-scale heterogeneity with increasing MTR score.

The MTR score was estimated on S1 using S1 as training data, but in a leave-two-subjects-out cross-validation fashion [10]: when scoring one subject, this subject as well as one randomly chosen subject of the opposite case–control status was left out of the training data. This methodology maintains the exact same number of cases and controls in the training set for all subjects scored, and thereby avoids any unnecessary bias.

MTR on S2 was scored using three different training algorithms: Training 1 (T1) used the S1 study as training data; Training 2 (T2) used the S2 study in a leave-two-out fashion as described for S1 above; Training 3 (T3) used the pooled S1 + S2 study in a leave-two-out fashion. The scoring using T1 was performed in Copenhagen and was blinded to outcome in S2. The MTR scores were transferred back to the Mayo Clinic for statistical analysis. Subsequently, case/control status was transferred to Copenhagen and T2 and T3 could be performed.

Projected mammographic breast area was computed based on the pectoral muscle line and the skin-air boundary. Inside this region, the distribution (histogram) of image intensities was recorded.

### Statistical analysis

Data presented are expressed as mean ± standard deviation unless otherwise indicated. Group characteristics were compared using the nonparametric two-sided Wilcoxon signed rank test. Reported confidence intervals are based on 95%. All tests were two-sided and considered significant when *P* <0.05.

A series of conditional logistic regression models with breast cancer as the outcome was fitted to each of the sets of training scores (T1 to T3). These models were adjusted for potential confounding variables: body mass index (BMI), menopause status, and postmenopausal hormone (PMH) use and percent density (PD). Odds ratios (OR) describe the association of the MTR scores with breast cancer, both as quartiles based on the control distribution and per one SD of each measure.

The ability to discriminate case vs. control status was evaluated as an area under the receiver operator characteristic (ROC) curve (AUC). AUCs were compared using the Delong test. For S2, AUCs were calculated within matched sets in order to utilize the matched nature of the sample. This was done by comparison of predicted model risk scores for cases and controls within matched sets and tabulating how often the case is correctly identified as having a higher risk score. Bootstrapping was utilized to provide confidence intervals for AUCs in this case.

Pearson correlation coefficients were used to summarize the association among different scores and PD measures. Kolmogorov-Smirnov tests were used to test differences in distribution of breast area and intensities.

## Results

Participants in S1 were older, mean, 58.0 ± 5.7 years, range (49 to 81), than participants in S2 (mean 55.2 ± 10.5 years, range (30 to 80) years). S2 had a longer time from mammogram to diagnosis than S1 (mean, 8.6 years, range (0.1 to 14.6) vs. mean, 3.7 years, range (2.2 to 4.2), respectively). In S2, controls were well-matched to cases on the majority of characteristics (Table 1).

**Table 1:** Characteristics of the cohort selected from the Mayo Mammography Health Study (S2)

**Study 2 characteristics**			
**Variable**	**Control (N = 442)**	**Case (N = 226)**	***P *** **value**
Patient age at date of mammography	54.8 ± 10.5	55.8 ± 10.6	0.28
Age			
50–59, No. (%)	125 (28%)	61 (27%)	0.73
60–69, No. (%)	108 (24%)	58 (26%)	0.73
70+, No. (%)	33 (7%)	22 (10%)	0.31
Body mass index (Kg/m^2^)	27.9 ± 6.6	27.9 ± 5.5	0.96
Postmenopause, No. (%)	277 (63%)	131 (58%)	0.24
Postmenopausal hormone use			
Never, No. (%)	226 (51%)	124 (55%)	0.36
Former, No. (%)	23 (5%)	9 (4%)	0.48
Current, No. (%)	119 (27%)	64 (28%)	0.70
Unknown, No. (%)	74 (17%)	29 (13%)	0.19
Time to diagnosis/enrollment	8.1 ±3.4	8.6 ±3.9	0.09
Breast area (cm^2^)	168.8 ±72.0	169.0 ±67.2	0.98

**Figure 2:**
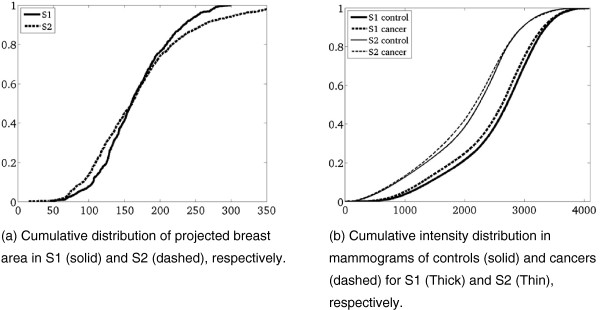
Distribution of mammographic breast area and mammographic pixel intensity in S1 and S2 respectively.

The mean projected breast area was significantly smaller in S1, a mean of 164.8 ± 49.7 cm^2^ compared to S2, a mean 168.9 ± 70.4 cm^2^ (*P* = 0.0016). The mean intensities also differed in the two studies, *P* <0.0001. Figure 2 shows the cumulative distribution of projected breast area and image intensities.

As reported previously [10], S1 showed a significantly (*P* <0.01) higher PD in the cases (22.3 ± 10.2%) than in the controls (19.7 ± 11.4%). Stratifying into screen-detected and interval cancers the density was higher (*P* <0.05) in those subsequently diagnosed as interval cancers (23.3 ± 1.0%) compared to screen-detected cancers (21.3 ± 0.9%) at the biannual screening visit. S2 similarly showed that cases had a higher mean density than controls (22.0 ± 15.4% vs. 18.4 ± 14.7%, *P* <0.01).

The association between quartiles of PD and breast cancer is shown in Table 2. As expected, there were more cases than controls in the higher quartiles, and fewer cases than controls in the lower quartiles for both studies; this is reflected in the ORs in Table 3.

**Table 2:** Stratification of subjects from S2 according to quartiles of controls based on various scores

**Score**		**Q1**	**Q2**	**Q3**	**Q4**
Percent density	Controls	109 (25%)	110 (25%)	110 (25%)	110 (25%)
	Cases	34 (15%)	65 (29%)	51 (23%)	76 (34%)
	*P* value	0.004	NS	NS	0.002
MTR	Controls	111 (25%)	111 (25%)	109 (25%)	111 (25%)
T1	Cases	38 (17%)	51 (23%)	50 (22%)	87 (38%)
	*P* value	0.015	NS	NS	<0.001
MTR	Controls	110 (25%)	111 (25%)	111 (25%)	110 (25%)
T2	Cases	31 (14%)	48 (21%)	66 (29%)	81 (36%)
	*P* value	<0.001	NS	NS	0.003
MTR	Controls	110 (25%)	111 (25%)	112 (25%)	109 (25%)
T3	Cases	35 (15%)	47 (21%)	63 (28%)	81 (36%)
	*P* value	0.005	NS	NS	0.002

Mammographic texture resemblance (MTR) scores require training. Numbers are given for training based on S1 (T1), S2 (T2), and S1 and S2 (T3) respectively. The latter two are performed in a leave-two-out fashion.

**Table 3:** Models for case/control status adjusted for body mass index (BMI), menopause age and postmenopausal hormone (PMH) use

**Model**	**AUC**	**OR per one SD**	**OR Q2**	**OR Q3**	**OR Q4**
Percent density	0.63	1.41 (1.16-1.72)	2.2 (1.3-3.7)	1.8 (1.0-3.1)	2.9 (1.6-5.2)
MTR T1	0.60	1.39 (1.17-1.66)	1.4 (0.8-2.3)	1.3 (0.7-2.2)	2.2 (1.4-3.6)
MTR T2	0.61	1.34 (1.14-1.59)	1.6 (0.9-2.7)	2.2 (1.3-3.9)	2.6 (1.5-4.3)
MTR T3	0.59	1.30 (1.10-1.54)	1.4 (0.8-2.4)	2.0 (1.1-3.7)	2.3 (1.4-3.9)
Percent density	0.66	1.36 (1.11-1.66)	2.2 (1.3-3.7)	1.8 (1.0, 3.2)	3.0 (1.6-5.5)
MTR T1		1.36 (1.13, 1.62)	1.0 (0.6-1.8)	0.9 (0.5-1.7)	1.8 (1.1-3.1)

Area under the receiver operator characteristic (ROC) curve (AUC) and odd ratios (ORs) per standard deviation (SD) are from the continuous model. The quartile analysis is from the discrete model with 95% confidence intervals. The last row includes the combined percent density (PD) and mammographic texture resemblance (MTR) T1 model AUC and contribution to OR for each of the parameters. The Model column contains four entities: MTR T1, MTR T2, MTR T3, and the combined PD and MTR T1 model.

The associations of MTR, for both quartiles and per SD, with breast cancer from all three training regimes are shown in Table 3. Adjusting for BMI, menopause, age and PMH use, all three trainings show similar associations between MTR and breast cancer and ability to discriminate case/control status (AUC 0.60 to 0.63).

Table 4 shows the correlation of MTR scores and PD. All training regimes of MTR show very high correlation (R >0.85, *P* <0.001) whereas correlation with PD is low (R <0.25).

**Table 4:** Correlation of the percent density and mammographic texture resemblance (MTR) scores in the three training regimes on S2

		**Percent density**	**MTR T1**	**MTR T2**	**MTR T3**
Percent density	Rho	1.00	0.03	0.12	0.22
	*P* value		0.44	0.003	<0.0001
	N	665	665	665	665
MTR T1	Rho	0.03	1.00	0.98	0.88
	*P* value	0.44		<0.0001	<0.0001
	N	665	668	668	668
MTR T2	Rho	0.12	0.98	1.00	0.90
	*P* value	0.003	<0.0001		<0.0001
	N	665	668	668	668
MTR T3	Rho	0.22	0.88	0.90	1.00000
	*P* value	<0.0001	<0.0001	<0.0001	
	N	665	668	668	668

Numbers are given for training based on S1 (T1), S2 (T2), and S1 and S2 (T3) respectively. The latter two are performed in a leave-two-out fashion. Reported are the Pearson correlation coefficient Rho, the *P* values of being different from 0, and N, the numbers of observations on which the estimates are based.

Finally, a combined model (Table 3, bottom) including both PD and the MTR score based on training on the independent cohort S1 (T1) show a slightly improved AUC of 0.66 ± 0.03 and a significant association of both PD and MTR with breast cancer risk. In comparison, a model including both PD and MTR on S1 yielded an AUC of 0.66 ± 0.02.

## Discussion

In numerous studies, including S1 and S2, PD, adjusted for age, BMI, and PMH use, has been associated with breast cancer risk [1-3]. In this and a previous study [10], the MTR score is also found to be a risk factor for breast cancer, that is, independent and complementary to the PD measure. In S2, MTR scores showed similar risk segregation capability, regardless of whether the MTR was realized, using training data from S1, S2, or a combination S1 + S2. This invariance to source of training data argues in favor of the robustness of the MTR score.

We found comparable associations and discrimination using the MTR in two different populations: the North American cohort (S2) was younger and had a wider range of age than the Dutch cohort (S1). As density in general decreases with age, the visual appearance of textural patterns may also be hypothesized to change by age. In fact, it was shown earlier that density invariant texture patterns may significantly separate randomly selected groups of 30 women differing five years in age [19]. However, given that the risk associations and discrimination did not differ between the MTR training regimes for the two studies, we may hypothesize that patterns important to risk are not changing drastically with age.

The mammographic technology also varied between the two studies, which used different film and digitizers. This serves as a potential source of noise in the recognition process of textures between studies. In Figure 2, the projected mammographic area and the intensity distribution are illustrated and shown to be significantly different. Notice especially that the intensities vary much more between studies than between cases and controls within studies. Hence, the study population and technology used for imaging make them appear significantly different. However, even with this variation in technology, the texture patterns were recognized across studies for their association to risk, underscoring the robustness of this measure.

The North American cohort has a larger projected breast area than the Dutch cohort. BMI measurements are present for the North American cohort while BMI was not recorded in the Dutch cohort. Breast size as cup size has shown to be inversely related to breast density measured on a different Dutch population [21], a trend that does not persist after correction for BMI and waist-to-hip ratio. Hence it may be interpreted that S1 has a lower BMI than S2. The density measured as a ratio of projected dense tissue to the projected breast size is in general inversely related to BMI, contributed mainly to the larger breast size whereas the dense area does not change with BMI [22]. Hence, we may hypothesize that the textures of dense tissue captured by MTR may therefore persist over ranges of BMI. This is still to be tested as BMI was not available on S1.

The differences in percent dense tissue between S1 and S2 may be somewhat explained by the large proportion of interval cancers in S1, the hypothesized variation in BMI, the differences in age, potential differences in PMH usage, and the interrater variation.

In S2 (Table 1), cancers and controls do not exhibit significant differences in the well-known risk factors of BMI and hormone usage. This may be partially contributed to by the age matching.

PMH use in the Dutch population was relatively low in the 1990s in the middle-aged population. Only 13 to 19% were current users with an average duration of two years [23]. In comparison, the North American cohort had 28% of current users. The Dutch cohort used for training (T1) is not necessarily balanced for hormone use in the cases versus controls. This shows that the rather dramatic effects PMH use may leave on parenchymal patterns [24,25] are not necessarily those picked up by the MTR methodology. Actually, those patterns that recognize combined estrogen and progestin treatment [25] are not present in different amounts in the controls and cases in S1 [10].

Time to diagnosis was considerably longer (8.6 years compared to 3.7 years) in the North American cohort S2. If patterns are stable and non-modifiable, the longer time window should only allow for more accurate outcome estimation, whereas if patterns change temporarily during the observation window, the longer time window could potentially contaminate prediction. As prediction is slightly (but insignificantly so) weakened in S2 one may hypothesize that texture patterns may potentially change over time, due to age, hormones, menopause, diet, and so on. Hence, it may be interesting to study the temporal variations of texture patterns in the individual.

The MTR shows a slightly weaker (0.61 compared to 0.63) but still significant capability to discriminate cases from controls in S2 compared to S1. This may be due to closer matching in S2 taking time interval prior to cancer into account, and not just age as in S1. In both S1 and S2, PD and MTR both persist as risk factors, showing that texture may carry additional recognizable information.

Measures such as fractal dimension [14] have been related to genetic status (BRCA1 and BRCA2). The associations between pixel intensity variance [12], Laws features, Markovian features, run-length features, Fourier features, wavelet features, and power-law features were compared on a case–control study by Manduca *et al*. [9]. Of these, the fractal dimensions, the Laws features, and the power-law features are all by design rotationally symmetric and do not differ for horizontal and vertical features. The Markovian, run-length, wavelet, and Fourier features potentially have the power to resolve anisotropic characteristics of the textures. Manduca *et al*. did find larger associations with coarse scale features. Figure 1 also seems to indicate that high MTR scores relate to the presence of large (coarse) scale textures. Manduca did not find significant improvement of the AUC by introducing any texture into models that included PD, which was not surprising given the correlation of the texture measures with PD (|R| = 0.39 to 0.76). The MTR scores show no or only weak correlation with PD (R = 0.03 to 0.22), and may thereby contribute more information to future breast cancer. In addition, unlike the texture features examined in Manduca *et al*. above, MTR features have the capability to distinguish spatially varying features (the indication of a pattern may vary with its position within the breast) and to measure aspects that were not intended for by design, as they are selected based on visual recognition capability, and not the mathematical design of features. This could also contribute to the differences in these two studies.

The cause and appearance of textural features relating to breast cancer risk is potentially very complex. Tissue density has been suggested to relate to altered protein composition and accumulation in the tissue, which may result in cancer [26]. This local deregulation may lead to an altered local extracellular matrix (ECM) environment relating to carcinogenesis [27,28]. This may well be understood as the components in the ECM not only anchor cells in proper spatial patterns, but also play important parts in regulating cell morphology, function, and apoptosis [28]. Mammographic density may therefore include effects of an altered matrix composition, in turn associated with carcinogenesis, whereas the tissue organization (MTR) may provide a score associated with local disorganization. The biological understanding of this being independent from matrix composition (density), may find support in other connective tissues and pathologies [29]. As tissue density and tissue distribution (MTR) were uncorrelated risk factors, this further supports that both accumulation and distribution are equally important for tissue function. Hence, both density and the spatial layout may contribute to risk assessment.

In fact, all three trainings of the MTR measure are highly correlated to each other, and at best weakly correlated to density. Also the MTR is associated with breast cancer, after adjustment for PD. This verifies the finding [10] that MTR complements the ability of mammographic density to discriminate those with and without future breast cancer.

The conclusions of the studies are limited to the demographics of the populations, including primary European populations. Furthermore, S1 was not matched or included risk factors other than age, and mammograms from the right side were always scored independently of the laterality [30] of future cancer. Samples including both pre- and postmenopausal women were analyzed together.

## Conclusions

We have shown that the mammographic texture resemblance, recorded in one study and examined in an independent cohort of different age distribution, geography, breast size distribution, X-ray and scanner technology, is a risk factor for breast cancer that is independent of percent density.

## Competing interests

Mads Nielsen has received grants from Nordic Bioscience A/S and holds shares in Biomediq A/S. Nico Karssemeijer holds shares in Matakina. Martin Lillholm holds shares in Biomediq A/S. Morten A Karsdal holds shares in Nordic Bioscience A/S. Celine M Vachon, Christopher G Scott, Konstantin Chernoff and Gopal Karemore declare that they have no competing interests.

## Authors’ contributions

MN took part in the overall design, instructed on the MTR scoring, and was the main writer of the manuscript including the discussion. CV and CS collected the S2 data, conducted the statistical analysis and contributed substantially to the writing of the manuscript. KC and GK contributed to the design of the MTR scoring, implemented the MTR scoring, conducted the MTR scoring and the data handling in Copenhagen, contributed to the discussion, and critically revised the manuscript. NK supervised the collection of the S1 data, contributed to the design of the study and the discussion, and critically revised the manuscript. ML contributed to the design of the MTR scoring, the statistical analysis, and the discussion, and critically revised the manuscript. MK contributed to the design of the study, provided insight on breast biology, wrote sections of the manuscript, contributed to the discussion, and critically revised the manuscript. All authors read and approved the final version of the manuscript.
